# Relationship Between Rhabdomyolysis and SARS-CoV-2 Disease Severity

**DOI:** 10.7759/cureus.53029

**Published:** 2024-01-27

**Authors:** Tijana Samardzic, Tinatin Muradashvili, Suzy Guirguis, Suleyman Felek, Samuel C Pan, Sandeep Tiyyagura, Richard Feinn

**Affiliations:** 1 Internal Medicine, Yale New Haven Hospital, Waterbury, USA; 2 Internal Medicine, Yale-Waterbury Internal Medicine Residency Program, Waterbury, USA; 3 Infectious Disease, Waterbury Hospital, Waterbury, USA; 4 Nephrology, Waterbury Hospital, Waterbury, USA; 5 Statistics, Frank H. Netter M.D. School of Medicine, North Haven, USA

**Keywords:** covid-19 mortality, covid-19 and aki, mortality rate in icu, rhabdomyolysis, covid-19

## Abstract

Background

Rhabdomyolysis has historically been associated with viral infections, of which influenza A is the most common. A literature review suggests that up to 1/3 of patients hospitalized with COVID-19 develop acute kidney injury (AKI), and of those, nearly half are admitted to the ICU. AKI complicating COVID-19 infection is attributed to several pathogeneses, including sepsis, direct cytopathic effects on the kidneys, and rhabdomyolysis.

Objective

We aimed to link COVID-19 infection to the development of rhabdomyolysis via creatine kinase (CK) measurement to assess whether this association increases ICU admission, length of stay (LOS), and mortality.

Design and setting

In this single-center, retrospective cohort study, we enrolled 984 adult patients with confirmed COVID-19 infection requiring admission to a community hospital between March 2020 and May 2021.

Measurements

Demographic data, laboratory values, and clinical outcomes were collected. The primary outcome measured was the development of rhabdomyolysis and/or AKI. Secondary outcomes included associations of rhabdomyolysis with ICU admission, length of hospital stay, and mortality, utilizing multivariable logistic regression methods.

Results

Out of the 984 patients included, 39 met the clinical criteria for rhabdomyolysis (4%). The incidence of rhabdomyolysis was higher in patients with AKI (38.3%) and in those who required ICU admission (53.8%) (p<0.001). There was an insignificant difference in death in this cohort (11 patients, 52.4%, p=0.996). However, the mean LOS in patients who had rhabdomyolysis was 18.2 days versus 9.8 days in patients who did not develop rhabdomyolysis (p<0.001).

Conclusion

Objectively tracking CK levels in COVID-19-infected patients can assist in diagnosing rhabdomyolysis, identifying AKI etiology, and accordingly making a preliminary prognosis for COVID-19 infection, which could direct physicians to initiate more intensive treatment earlier.

## Introduction

With over 750 million cases of SARS-CoV-2 infection confirmed globally, the COVID-19 pandemic continues to be a focus of research due to many unknown features of the virus and long-term patient outcomes [1-3]. Symptoms can range from mild illness to severe deterioration requiring ICU-level care. It is crucial to better understand the complications of the disease to predict severity and tailor management plans. One such complication of COVID-19 is the development of rhabdomyolysis in conjunction with acute kidney injury (AKI), which is not yet well understood.

Rhabdomyolysis is characterized by the breakdown of skeletal muscle and the resultant release of intracellular contents into the bloodstream, leading to systemic complications, including AKI [4]. Viral infections, historically influenza A, are a known cause of rhabdomyolysis [5]. Emerging literature has also linked the syndrome to the SARS-CoV-2 infection [6-8]. Research has suggested that certain inflammatory markers, including C-reactive protein (CRP), erythrocyte sedimentation rate (ESR), ferritin, interleukin-6 (IL-6), interleukin-8 (IL-8), procalcitonin, and serum amyloid A, may be used to monitor the severity of COVID-19 infection and its progression toward multi-organ involvement [9]. Additionally, creatine kinase (CK), a known indicator of rhabdomyolysis and the development of AKI, has been observed to be increased in COVID-19 patients [7]. Rhabdomyolysis is diagnosed by the presence of myoglobinuria in addition to an elevated CK U/L ≥1000 [4].

Current information surrounding COVID-19 and rhabdomyolysis has mainly been published in case reports. The limited data have suggested that up to 1/3 of COVID-19 patients who are hospitalized develop AKI [7], and of those, nearly half are admitted to the ICU [10-12]. The link to rhabdomyolysis is not straightforward, since it can develop at the start of the COVID-19 illness or may occur at any point along the course of the disease [13] and may be difficult to characterize. Patients with COVID-19 and rhabdomyolysis have a higher risk of progressing to a critically severe infection, requiring more ICU admissions, higher rates of the requirement for mechanical ventilation, and increased mortality [14]. Furthermore, although the standard of care for patients with rhabdomyolysis is to administer fluids aggressively, COVID-19 patients are at risk of acute hypoxemic respiratory failure from fluid overload. Thus, managing COVID-19 patients with rhabdomyolysis is complicated, and fluids must be administered cautiously [15]. Clinicians must be aware of the potential for COVID-19 patients to present with rhabdomyolysis or develop it along the disease course to develop proper treatment regimens that can be initiated early.

More information on the relationship between COVID-19 and rhabdomyolysis is needed. Objectively tracking the manifestation of rhabdomyolysis in COVID-19-infected patients can help guide an effective treatment and prevent progression to acute renal failure. This retrospective analysis aims to find a link between COVID-19 infection, increased CK levels, and the development of acute renal failure to add to the current body of literature and identify a means to recognize early deterioration of the infection.

## Materials and methods

A retrospective chart review was conducted on 984 patients over the age of 18 who were admitted to Waterbury Hospital, a 357-bed community medical center, from March 2020 to May 2021. All patients tested positive for SARS-CoV-2 through RT-PCR analysis of nasal or nasopharyngeal swab samples. The study received approval from the Institutional Review Board of Waterbury Hospital (approval number: 20-022). Patients who were pregnant or discharged on the same day were excluded from the study. Demographics, body mass index (BMI), and laboratory results, specifically for urinalysis (UA), the maximum level of CK, aldolase, and lactate dehydrogenase (LDH), were documented.

To diagnose chronic kidney disease (CKD) and AKI, we used serum creatinine (Cr) trending graphs during data collection. The diagnosis of AKI was based on KDIGO (Kidney Disease: Improving Global Outcomes) criteria, with patients having an increase in serum Cr of ≥0.3 mg/dL within 48 hours or ≥50% within seven days being defined as having AKI [16]. Urine outputs were not extracted and, therefore, not used for AKI diagnosis. We defined rhabdomyolysis as the presence of CK U/L ≥1000 along with the presence of myoglobinuria. We compared CK levels and AKI incidence to determine if rhabdomyolysis caused AKI in patients with a severe COVID-19 infection. We also identified patients’ characteristics and assessed for associations with the following: diagnosis of rhabdomyolysis, ICU admission, mortality, and length of stay (LOS).

The primary outcomes analyzed were the association of rhabdomyolysis with AKI during acute COVID-19. The secondary outcomes were the association of rhabdomyolysis with ICU admission, increased LOS, and mortality. Unadjusted analysis was performed using Pearson Chi-square and Fisher's exact test, while logistic regression was used for multivariable analysis. All analyses were conducted using SPSS Statistics version 28 (IBM Corp. Released 2021. IBM SPSS Statistics for Windows, Version 28.0. Armonk, NY: IBM Corp.). The alpha level for statistical significance was set at 0.05.

## Results

During the study period of December 2019 and March 2021, a total of 984 adult patients were admitted with COVID-19 infection to Waterbury Hospital. The demographics and comorbidities are reported in Table 1. The patients included 513 (52%) males and 467 (48%) females. The average age was 64 ± 17.6 and the mean BMI was 31.3 ± 9.2. Comorbidities included diabetes mellitus (DM; 341 (34.7%)) and CKD (196 (19.9%)).

**Table 1:** Demographics, clinical characteristics, and comorbidities BMI: body mass index, CK: creatine kinase, LDH: dehydrogenase, CKD: chronic kidney disease, DM: diabetes mellitus, LOS: length of stay

Data item	Total (N (%) or mean ± SD)
Average age (years)	63.81 ± 17.59
Average BMI	31.3 ± 9.2
Average LOS (days)	9.84 ± 16
Male	513 (52%)
Female	467 (48%)
Average CK (IU/L)	1181 ± 12153
Average LDH (U/L)	1107 ± 757
Total with CK ≥1000 (IU/L)	63 (12.4%)
Total with myoglobinuria	244 (24.8%)
History of CKD	196 (19.90%)
History of DM	341 (34.70%)

**Figure 1:**
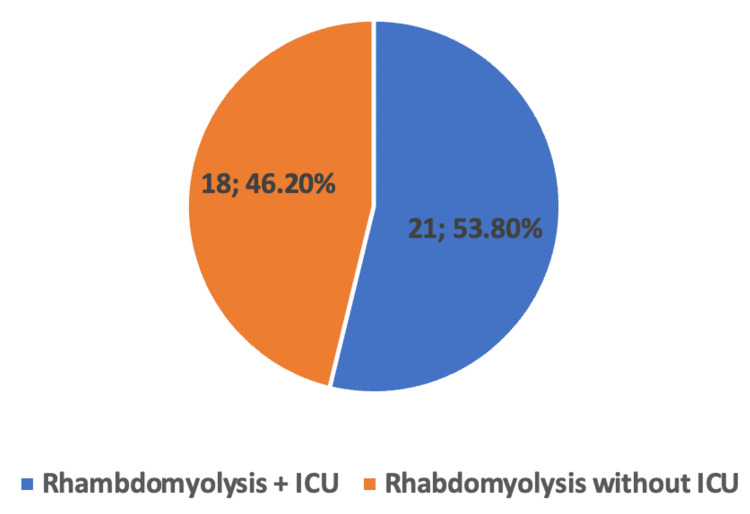
Pie Chart diagram displaying percentages of patients who had rhabdomyolysis and required admission to the ICU ICU: intensive care unit

Out of the 984 patients, 39 (4%) were diagnosed with rhabdomyolysis. Out of the total 204 (20.9%) patients who were admitted to ICU, 21 (10.3%) had rhabdomyolysis. Of note, CK levels were not drawn in 229 patients and UA was not obtained in 476 patients thereby contributing to possible underdiagnosis of rhabdomyolysis, although CK levels remain to be the most sensitive diagnostic measure. Out of the patients who had a diagnosis of rhabdomyolysis, 27 also had AKI (69.2%, p<0.001), 21 patients were admitted to the ICU (53.8%, p<0.001), and 14 suffered death (35.9%, p<0.002) (Table 2, Figure 1). The mortality rate for the patients with rhabdomyolysis requiring ICU care was not found to be statistically significant compared to those without requiring ICU care.

**Table 2:** Primary outcomes: development of rhabdomyolysis and various measures BMI: body mass index, ICU: intensive care unit, AKI: acute kidney injury

	Patients with rhabdomyolysis	All Patients	Fisher's exact test (2-sided p-value)
Number of patients	39 (4%)	984	
Average age (years)	61 ± 17.9 SEM 2.8	63.81 ± 17.6	0.212
Average BMI	35 ± 11.3 SEM 1.8	31.32 ± 9.2	<0.006
ICU admission	21 (53.8%)	206 (21%)	<0.001
AKI	27 (69.2%)	382 (39%)	<0.001
Death	14 (35.9%)	158 (16%)	<0.003

**Figure 2:**
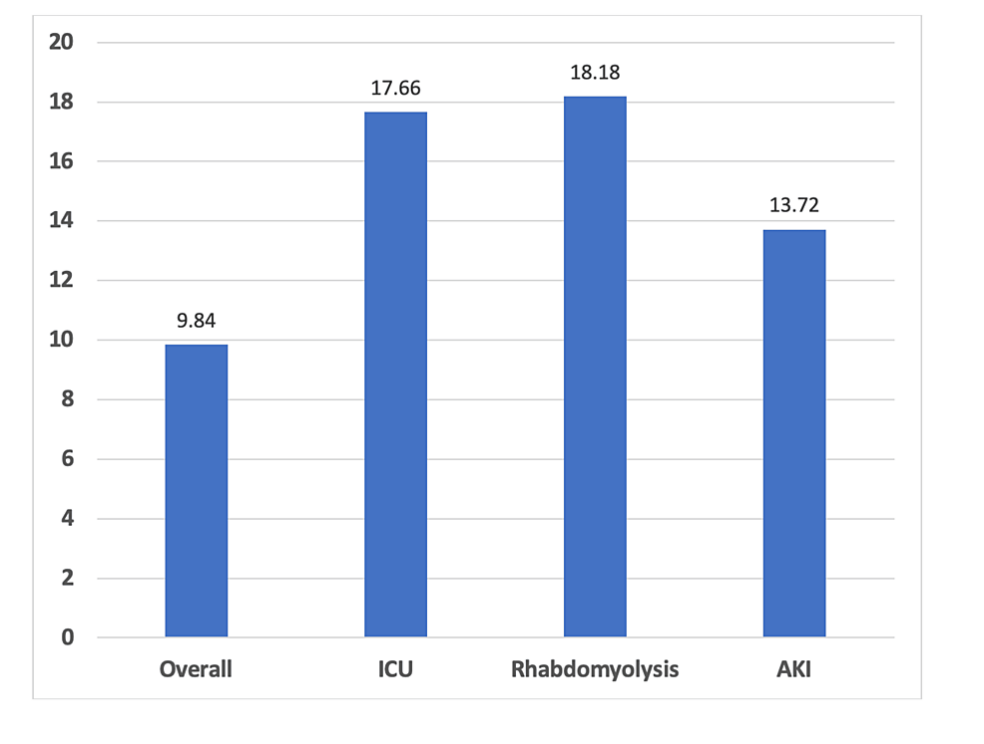
Bar diagram indicating the average LOS (days) ICU: intensive care unit, AKI: acute kidney injury, LOS: length of stay

The average LOS among all patients was 9.84 ± 16 days. LOS was significantly higher in patients with rhabdomyolysis (18.18 days, (p<0.001), and in patients with AKI (13.72 days, p<0.001) (Figure 2).

Overall, the project revealed increased LOS and the requirement for ICU admission when suffering from rhabdomyolysis. AKI and rhabdomyolysis were highly linked, and there was a significant need for ICU stays with these diagnoses present. Death was not found to be statistically different between those with rhabdomyolysis and those who required the ICU or not (with: p=0.966; without: p=0.242); however, compared to all patients, death was significant in those with rhabdomyolysis. Interestingly, when comparing mortality rates in patients with rhabdomyolysis to mortality rates in AKI, AKI had a more significant relation to death (OR 4.566 vs. 3.115).

## Discussion

The COVID-19 pandemic has posed a significant public health threat that has resulted in high mortality rates and long-lasting side effects, especially in cases of severe disease. Therefore, identifying ways to predict disease severity is crucial to guiding interventions and providing more intensive treatment upfront. Rhabdomyolysis is the consequence of the rapid breakdown of skeletal muscle tissue in various conditions, including trauma, hyperthermia, intense exercise, drugs, toxins, electrolyte disorders, or infections [4]. The presence of rhabdomyolysis is diagnosed by Cr kinase levels, typically above 1000 units, and may be associated with myoglobinuria, muscle weakness, and cramps. Without rapid and aggressive treatment with intravenous fluids, rhabdomyolysis can lead to severe multiorgan injury, particularly to the kidney and liver. Several viruses have been linked to the consequences of this disease, and the link between this disease and the SARS-CoV2 virus has been shown in several case reports [4,8,13-15]. While a meta-analysis done in 2020 revealed an association between worsening kidney disease and death in COVID-19-infected patients, the presence of rhabdomyolysis was not measured, despite its association with kidney injury and acute viral illness [12].

This study aimed to establish a correlation between rhabdomyolysis and the severity of COVID-19 illness, defined by increased LOS, ICU admission, and mortality. The results showed that nearly 70% of COVID-19-infected patients who developed AKI also met the criteria for rhabdomyolysis (p<0.001). Of these patients, more than half required an ICU admission and experienced an increased hospitalization LOS (p<0.001), highlighting that organ failure, specifically kidney failure, is at least partially related to the severity of the COVID-19 illness. Previous publications have suggested that rhabdomyolysis is associated with a higher risk of progression to severe infection, more ICU admissions, higher rates of mechanical ventilation, and increased mortality [8,11,12].

This study has several limitations that should be considered when interpreting the results. The study was conducted early in the COVID-19 pandemic; therefore, several patients were likely not tested for SARS-CoV2, limiting our sample size. Additionally, many patients did not undergo testing for CK, which is necessary for the proper diagnosis of rhabdomyolysis. This may have limited the incidence of rhabdomyolysis observed in this study. Many patients also did not undergo testing for UA for the presence of myoglobinuria; however, its use is very limited in the diagnosis of rhabdomyolysis.

Moreover, all patients included in the study were admitted to a single community hospital, contributing to our low sample size, which may not represent a diverse or severely ill population compared to larger healthcare systems. Thus, more research is required on different populations to draw more generalizable conclusions. Additionally, it may be worth studying the time of onset of rhabdomyolysis throughout the course of severe COVID-19 disease as well as assessing for other contributors to the development of rhabdomyolysis.

## Conclusions

SARS-CoV2 disease can lead to multiorgan failure through multiple mechanisms, thereby contributing to severe disease, as defined by high morbidity and mortality, ICU level of care, and subsequent increased LOS. Despite the limitations listed, the study adds to the current understanding of the relationship between COVID-19 infection, rhabdomyolysis, and AKI, suggesting that objective measurement of CK levels is compulsory to assess the severity of SARS-CoV2 and allow for the opportunity for earlier intervention.

Further research is needed to validate these findings in larger and more diverse populations to better understand the mechanisms involved in severe COVID-19 infection, guide clinical interventions, and aid in preventing severe morbidity and mortality in those suffering from rhabdomyolysis.
